# The effect of an anti-inflammatory diet on chronic pain: a pilot study

**DOI:** 10.3389/fnut.2023.1205526

**Published:** 2023-07-13

**Authors:** Marta Sala-Climent, Teresa López de Coca, María Dolores Guerrero, Francisco Javier Muñoz, María Amparo López-Ruíz, Lucrecia Moreno, Mónica Alacreu, María Auxiliadora Dea-Ayuela

**Affiliations:** ^1^Cátedra DeCo MICOF-CEU UCH, Universidad Cardenal Herrera-CEU, CEU Universities, Valencia, Spain; ^2^Department of Pharmacy, Universidad Cardenal Herrera-CEU, CEU Universities, Valencia, Spain; ^3^Department of Mathematics, Physics and Technological Science, Universidad Cardenal Herrera-CEU, CEU Universities, Valencia, Spain

**Keywords:** pain, anti-inflammatory diet, chronic pain, rheumatic diseases, pro-inflammatory foods

## Abstract

**Objective:**

Rheumatic diseases result in chronic pain (CP) and require treatment with drugs whose prolonged administration is associated with side effects. However, publications in the academic literature have suggested that diet modification and food supplementation can play a crucial role in alleviating the symptoms of inflammatory disease. Thus, it is hoped that the use of an anti-inflammatory diet for pain management might result in improved quality of life. Hence, here we aimed to investigate the effect of anti-inflammatory foods in patients with CP caused by rheumatic diseases.

**Methods:**

After an exhaustive bibliography search, we designed a 13-item anti-inflammatory dietary guide based on a Mediterranean diet without red meat, gluten, or cow’s milk (the AnMeD-S). We then conducted a pilot study to evaluate the efficacy of this anti-inflammatory diet in patients with CP. A food consumption score (with a maximum of 156 points) was then applied to evaluate patient adhesion to the proposed diet. Forty-five patients with CP were followed-up for 4 months. Variables related with quality of life (including pain perception, depression status, and sleep satisfaction) were measured using 9 validated questionnaires and anthropometric measurements were recorded before and after the participants followed the anti-inflammatory diet.

**Results:**

We found a correlation between increased anti-inflammatory food intake and improved physical characteristics, stress, and pain in the patients we assessed. Moreover, decreased consumption of pro-inflammatory foods was positively correlated with sleep satisfaction. Following the AnMeD-S was associated with improved physical characteristics and quality-of-life in patients with CP.

**Conclusion:**

The AnMeD-S, includes anti-inflammatory foods and restricts the consumption of certain pro-inflammatory foods (such as those containing gluten). This dietary pattern could provide relief from CP and improve the symptoms of stress and depression, as well as reducing sleep disturbances.

## 1. Introduction

Pain is described as one of the most uncomfortable feelings a patient can experience. According to the International Association for the Study of Pain there are three main categories of chronic pain (CP). First, nociceptive pain is caused by tissue damage and is the most common form of CP. Second, neuropathic pain is caused by damage to or diseases affecting the somatosensory nervous system such as neuropathies, stroke, and multiple sclerosis. Third, nociplastic pain arises from abnormal pain signaling processes in the absence of evidence of any related tissue damage or pathologies. It is caused by neuroplasticity of nociceptive or neuropathic pain (1). Among the causes of CP, rheumatic diseases have a high prevalence. According to the World Health Organization (WHO), 20% of the world population suffers from some form of rheumatic disease and they account for 35% of the causes of overall disability in the adult population of the Western world (2).

CP in rheumatic diseases such as rheumatoid arthritis (RA), fibromyalgia (FM), and osteoarthritis (OA) has been shown to have a significant bidirectional relationship with several psychological, cognitive, and social factors such as depression, anxiety, stress, or poor sleep (3, 4). More recently, other factors that should be considered in the management of CP, such as poor diet and being obese/overweight, have received more attention (5, 6), with some studies suggesting that elevated body fat may infer an increased risk of medical incidents and worsening joint pain (7–9). The bidirectionality of the symptomatology of these pathologies means that there is feedback between them and so, improving these symptoms could also improve all the comorbidities mentioned above. Moreover, this bidirectionality might be influenced by the gut microbiome and increased permeability of the gut barrier, thereby increasing neuroinflammation and accelerating neurodegeneration (10). Of note, the chronic systemic inflammation caused by rheumatic pathologies could cause this increased permeability (6). Dietary interventions to improve symptoms are common in these patients (7) and in this sense, a wide range of studies have shown promising results when patients with rheumatic diseases have followed an anti-inflammatory diet (3, 8, 11).

The standard western diet is characterized by high levels of consumption of saturated fats, refined carbohydrates, red meat, salt, and sweetened beverages, and nowadays this style of eating has extended worldwide (12). Adherence to a western diet can cause excessive production of pro-inflammatory mediators along with the reduced release of anti-inflammatory mediators, including antioxidants. In fact, together with inflammation, a lower intake of micronutrients such as omega-3 fatty acids, vitamins B and D, magnesium, zinc, and B-carotenes is associated with CP (13). Given this, and based on a large body of scientific knowledge, the WHO (2) and other scientific institutions now recommend the Mediterranean diet (MD) as the healthiest style of eating because it can reduce the risk of several chronic diseases. The MD is characterized by high consumption of fruits and vegetables, extra virgin olive oil (EVOO), nuts, legumes, unprocessed cereals, and fish; moderate consumption of dairy products and meat; and low consumption of red meat and ultra-processed foods.

Of note, based on the composition of the MD, it is classified as an anti-inflammatory diet. Importantly, this type of regimen is of key interest in many chronic diseases. Recent studies have shown encouraging results in patients with CP after nutritional interventions (7, 14–18) thanks to decreased intestinal barrier permeability as the result of lower pro-inflammatory mediator production, thereby preventing the passage of certain metabolites into the bloodstream (19, 20). In addition, the consumption of anti-inflammatory foods seemed to improve patient quality of life (QoL) by decreasing their levels of stress, anxiety, depression, and cognitive and sleep disturbances (21), achieved by reducing inflammation and oxidative stress (22).

While MD, has shown promising results in mitigating inflammation and enhancing patient well-being, additional measures may be warranted to optimize therapeutic outcomes. Turmeric, derived from the *Curcuma longa* plant and rich in the bioactive compound curcumin, has emerged as a potential coadjuvant supplement in anti-inflammatory dietary strategies (23). Curcumin possesses anti-inflammatory properties and can modulate multiple pathways involved in inflammation, thereby potentially providing additional benefits to rheumatic patients.

Thus, starting from this hypothesis, in this current work our objectives were to summarize the current academic knowledge of anti-inflammatory and pro-inflammatory foods in order to design a nutritional guide and to evaluate the use of this anti-inflammatory dietary intervention in patients with CP caused by rheumatic diseases to establish the efficacy of this guide to improve the QoL of these patients. In addition, this pilot study also aims to explore the inclusion of turmeric supplementation with curcuma latte (Baïa Food^®^) as a coadjuvant to the anti-inflammatory diet, with a focus on its potential synergistic effects in alleviating pain, reducing inflammation, and improving overall patient outcomes.

## 2. Methods

### 2.1. Search strategy to determine the basis of the anti-inflammatory diet

In the first part of this study, we set the bases for the anti-inflammatory diet we would use in the second part of the work. Thus, we performed an exhaustive search to identify anti-inflammatory diets and nutrients described in the academic literature; we also performed a search for pro-inflammatory foods. The search was conducted from 1 November 2020 to 30 January 2021 using “anti-inflammatory diet,” “chronic pain,” and “rheumatic diseases” as the key words, although other key words were also used to better understand certain foods or nutrients such as glutenin or casein thought to be involved in inflammation. We only considered articles written in English and studies carried out on humans aged over 45 years.

### 2.2. Study participants

Forty-five patients were recruited from different Valencian Rheumatoid Patient Associations, with permission of the presidents of each association. All the patients had previously been diagnosed by rheumatologists and were all treated after signing their informed written consent to participation in this work. This study was reviewed and approved by the Institutional Review Board (IRB) at the CEU Cardenal Herrera University (CEEI20/095, approval date: 31 March 2021) and was compliant with the European General Data Protection Regulation (RGPD) and Organic Law 3/2018 on the Protection of Personal Data and the Guarantee of Digital Rights which guarantees data protection and data security, confidentially, and use for the purpose for which the participants were properly informed. Moreover, the study complied with the principles of the Declaration of Helsinki.

### 2.3. Intervention

**Figure 1:**
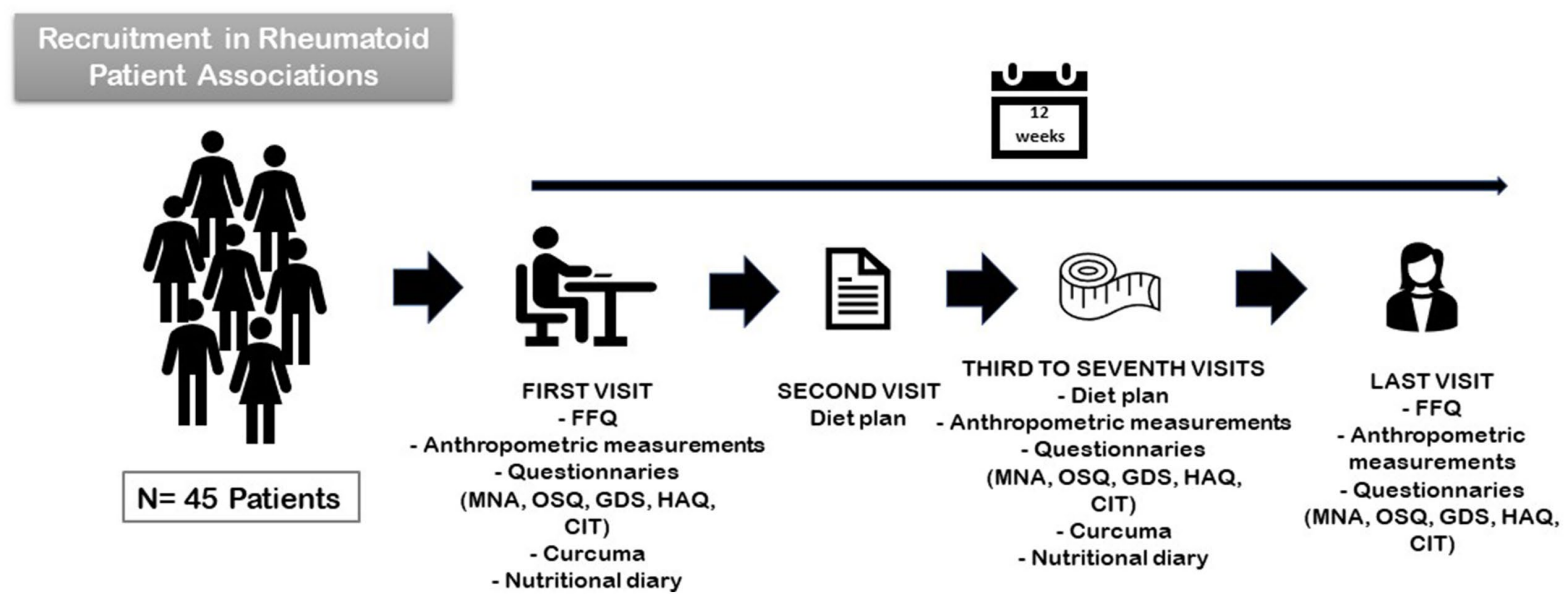
Nutritional intervention study with an anti-inflammatory, supplemented MD diet.

We conducted a 4-month nutritional intervention study with an anti-inflammatory, supplemented, MD (AnMeD-S) in which pro-inflammatory foods such as gluten products, cow’s milk, red meat, alcohol, sugar, and processed foods were removed. We also recommended an increase in anti-inflammatory foods such as blue fish, EVOO, nuts, fruits, vegetables, legumes, plain yogurt and kefir, and turmeric. Additionally, each month we provided the patients with a container of *Curcuma Latte* (Baïa Food^®^) which contains curcuma and black pepper to enhance curcumin absorption. The curcuma powder was given to the patients because it is not usually consumed as part of the MD. The study consisted of 5 face-to-face and 5 telematic visits with in-person visits scheduled once a month and the first telematic visit being scheduled 15 days after the first in-person visit and monthly thereafter. Moreover, Zoom^®^ meetings (Zoom Video Communications, Inc.) were also programmed every month to provide dietary education (Figure 1).

#### 2.3.1. First visit

The study was explained to the patient and the informed consent was then signed. All the participants completed a 24-h food recall and a food frequency questionnaire (FFQ) which were used in the preparation and planning of individual menus. Moreover, during the examination, anthropometric measurements were taken and the Mini Nutritional Assessment (MNA) (24), Oviedo Sleep Questionnaire (OSQ) (25), Geriatric Depression Scale (GDS) (26), Health Assessment Questionnaire (HAQ) (27, 28), and Cognitive Impairment Test were completed (29–31). At the end of the visit, the nutritionist gave each patient a container of *Curcuma Latte* and a nutritional diary; the latter was used to record the daily food intake. Five days later, a weekly menu, recipes, and a list of seasonal fruits was sent to each patient by e-mail.

#### 2.3.2. Second visit

The diet plan was customized to suit each patient’s circumstances, preferences, and beliefs to encourage adherence. Fifteen days later the dietitian called each patient to assess how they were adhering to the diet, what parts were easier/harder for them to follow, and to adjust the diet if necessary.

#### 2.3.3. Third visit

One month later, the patients met face-to-face with the study dietitian to receive counseling. The patients’ anthropometric measurements were recorded, and they completed the HAQ, GDS, and OSQ questionnaires. Each patient received another pack of *Curcuma Latte*, a new nutritional diary, another monthly menu, recipes for the menu, and a list of seasonal fruits and vegetables.

#### 2.3.4. Fourth to eighth visit

From first month until the end of the study the patients were asked to meet face-to-face once a month so that we could record their anthropometric measurements, complete the questionnaires, and provide the monthly menu, *Curcuma Latte*, and nutritional agenda. Patients received biweekly phone calls to check on their progress, answer questions, and complete questionnaires. Every month, they were also offered the chance to attend a support group facilitated by our dietitian, either on Zoom^®^ or face-to-face, which was designed to reinforce the dietary interventions.

#### 2.3.5. Last visit

Four months after starting the intervention, the patients met face-to-face with the dietitian and completed all the aforementioned tests and anthropometric measurements for the last time.

The intake of pro-inflammatory and anti-inflammatory foods was recorded by the patients daily; we also asked about physical exercise, stress, pain, and sleep quality every day. The patients could stop the dietary intervention upon request. At the end of the study (4 months), the diets followed by each patient were documented and collated so that all of the data could be analyzed in conjunction.

### 2.4. Nutritional questionnaires

Different questionnaires were used to evaluate the nutritional status of each patient. First, an adaptation of the Predimed FFQ was used at the beginning and end of the study to determine the evolution of the participants and frequency of the foods consumed (32). In addition, the MNA questionnaire (24) was used to rate the patients’ risk of malnutrition.

### 2.5. Pain, insomnia, stress, and depression questionnaires

We used the OSQ (25) to assess sleep disorders. Perceived pain was evaluated with a numeric rating scale (NRS) (33), patient disability was estimated with the HAQ (27, 28), and depression with the GDS (26). Moreover, the same NRS was employed to measure stress in order to compare the participant’s perceptions of pain, rest, and stress (33).

### 2.6. Cognitive impairment questionnaires

A cognition screening was performed at the beginning and end of the 4-month intervention using the Memory Impairment Screening (MIS) (29), Short Portable Mental State Questionnaire (SPMSQ) (30), and Semantic Verbal Fluency (SFV) (31) tests. The characteristics of the 9 validated questionnaires used to measure nutrition status, cognitive impairment, pain, insomnia, and depression are shown in Table 1.

**Table 1:** Summary of the characteristics of the validated questionnaires used in this study.

Characteristic	Validated tests	Reference value		
Nutrition	Mini Nutritional Assessment (MNA)	[0–7] Malnutrition	[8–12] Malnutrition risk	[12–14] Well nourished
Pain	Numeric rating scale (NRS)	[0–2] Light	[3–7] Moderate	[8–10] Intense
Stress	NRS	[0–2] Light	[3–7]e Moderate	[8–10] Intense
Depression	Geriatric Depression Scale (GDS)	[0–9] Normal	[9–20] Symptoms compatible with low to moderate depression	[20–30] Symptoms compatible with moderate to severe depression
Disability	Health Assessment Questionnaire (HAQ)	[0–1] Mild disability	[1, 2] Mild to moderate disability	[2, 3] Severe disability
Sleep quality	Oviedo Sleep Questionnaire (OSQ)	[1–7] Subjective sleep satisfaction	Low values indicate sleep problems	
		[1–15] Hypersomnia	High values indicate sleep problems	
		[1–45] Insomnia	High values indicate sleep problems	
Cognitive impairment	Semantic Verbal Fluency (SFV)	<10 symptoms compatible with DC		
	Memory Impairment Screen (MIS)	<4 symptoms compatible with DC		
	PFEIFFER’s Short Portable Mental State Questionnaire (SPMSQ)	>3 symptoms compatible with DC		

### 2.7. Diet score

To evaluate adhesion to our proposed anti-inflammatory diet, a modified version of the ITIS diet score (34) created by the University of California San Diego was adjusted to the AnMeD-S. A total score of 156 points was possible using our scoring system. Negative points being assigned if refined grains, red meat, more than 4 eggs per week, saturated fats, milk, flavored yogurts, more than 3 coffees per day, alcohol, sweetened beverages, sugar, pastries, or cakes, or sauces such as ketchup and mayonnaise, etc. had been ingested. From among the remaining foods evaluated, the most important ones such as turmeric, EVOO, blue fish, nuts, dark chocolate, fruits, and plain yogurt or kefir received more points. Table 2 shows the overall scoring system and how it was calculated based on how the patients answered the FFQ.

**Table 2:** Foods included in the anti-inflammatory, supplemented, Mediterranean diet and their recommended consumption frequencies.

Food	Importance	Consumption frequency	References
Extra virgin olive oil (EVOO)	EVOO is rich in antioxidants, especially phenolic compounds. The beneficial effects of EVOO are associated with its fatty acid composition, which is very rich in monounsaturated fatty acids, with moderate levels of saturated fatty acids and polyunsaturated fatty acids (PUFAs).	3 tablespoons/day	(16, 38, 39)
Fruits	Rich in polyphenols. Different colored fruits are especially useful because of their carotenoid content anti-inflammatory properties.	3 portions/day	(3)
Nuts	Rich in monounsaturated fatty acids (MUFAs) and many minerals and vitamins.	30 g daily	(39, 41)
Vegetables	Rich in antioxidants such as flavonoids, carotenoids, phenolics, isoflavones, and indoles, as well as vitamins. Contain high levels of fiber.	2 portions/day	(42)
Plain yogurt/kefir	Regulation of intestinal flora and pro-inflammatory cytokines in the gut.	1 portion/day	(38)
Coffee	Rich in phenolic compounds including chlorogenic acids (CGAs), which reduce pro-inflammatory cytokines such as TNF-α.	<3 cups/day	(43, 44)
Eggs	Contains fat-soluble B vitamins, minerals, choline, and carotenoids.	3–4/week	(45)
Blue fish	Rich in fatty acids and ω-3 PUFAs.	3 portions/week	(36, 39)
Legumes	Rich in fiber, minerals and in vitamins. Soybeans reduce the presence of pro-inflammatory cytokines.	3 portions/week	(42)
Red meat	Reduced consumption appears to be associated with improvements in RA symptoms.	Occasionally	(39, 46)
Cow’s milk	Rich in trans-fatty acid which increases the presence of inflammatory markers. Moreover, the saturated fatty acids present in cow’s milk increase inflammation.	Avoid completely	(4)
Gluten	The gliadin and glutenin contained in gluten both trigger immunological responses.	Avoid completely	(8)
Curcumin	Inhibits the expression of pro-inflammatory cytokines (IL-1, IL-8, and IL-6) and TNF-α.	Every day in conjunction with black pepper^†^	(4)

This table is provided only as an orientation for the anti-inflammatory diet the study participants we followed; all the patients’ menus were specifically adjusted for their preferences and their recommended energy consumption in terms of calorific intake. ^†^Black pepper enhances curcumin absorption.

We separated the vegetable score into three groups. The first group was greens and included lettuce, spinach, chard, endive, green beans, and other types of green leafy vegetables. The second group was of non-greens and included tomato, asparagus, celery, peppers, artichoke, mushrooms, and carrots. The third group contained every other vegetable including cauliflower, broccoli, aubergine/eggplant, courgette/zucchini, cucumber, brussels sprouts, and other commonly used vegetables. In addition, the fruits were also separated into three groups. The first group contained berries including strawberries, grapes, fruits of the forest (raspberries, blackberries, and blueberries, etc.), and cherries. The second group contained enzymatic fruits including orange, tangerine, grapefruit, pineapple, kiwi, and banana. The last group included other fruits such as apple, pear, peach, melon, and watermelon. After evaluating the score, we rated them as percentages.

### 2.8. Variables studied

To evaluate the therapeutic effect of the AnMeD-S we collected the following data:

- Demographic variables: age, gender, chronic pain, diagnosis of depression, and family background.
- The AnMeD-S variables at the beginning and end of the follow-up, applying the following scores: healthy diet [0–60 points], anti-inflammatory foods [0–96 points], negative score for pro-inflammatory foods [−66–0 points], total anti-inflammatory diet score [0–156 points]. After the start of the study, some new variables were considered such as percentage consumption of a healthy diet ([0–100%]), anti-inflammatory foods ([0–100%]), pro-inflammatory foods (based on the highest score achieved by the patients; [0–100%]), and global percentage of the anti-inflammatory diet adherence ([0–100%]).

Likewise, we also calculated some new variables to rank the patients into different groups. For each monitored food, the first group were patients already following the recommendations at the start and end of the study (“maintain”). The second group were patients that improved their diet to meet our recommendations (“meet”). The third group were the patients who improved their diet but did not meet our recommendations (“improve”). The last group were patients who maintained their habits and did not meet our recommendations or whose diet had worsened (“worsen”).

The classification remained as followed: consumption of a healthy diet, consumption of anti-inflammatory foods, consumption of pro-inflammatory foods, and finally, overall consumption of an anti-inflammatory diet (“low”: [0–50%]; “moderate”: [50–75%]; and “high”: [75–100%]).

- Physical condition variables: weight, body mass index (BMI), body fat (BF), body water (BW), muscle mass (MM), metabolic age (MA), visceral fat (VF), arm circumference (AC), waist circumference (WC), hip circumference (HC), and waist-hip index (WHI). From the follow-up of these variables, we built new variables that classified patients according to whether they improved each of these physical metrics. All the variables were measured as an improvement if the result had decreased, except for muscle mass and undernutrition, which were noted as improved if they had increased.
- QoL variables: measured using the validated questionnaires. New variables are made based on the follow-up to classify the patients as having improved or not for each characteristic.

### 2.9. Statistical analysis

The sample size was calculated to ensure that the work would be able to detect a change in the food consumption based on the score and a reduction in the degree of pain after the dietary intervention. Therefore, the sample size was determined to maximize these two conditions as follows:

To detect the effect of increased consumption of the AnMeD-S with a confidence of 95% and statistical power of 80%, taking a mean increase of 17% and a standard deviation of 20% as reference values, a sample size with more than 11 individuals was required.

To determine the effect of a reduction in the degree of pain by a mean of 1 point and a standard deviation of 2 points, with a confidence of 95% and a statistical power of 80%, a sample size exceeding 27 participants was needed.

Finally, we recruited a total of *n* = 45 participants to this study, which therefore exceeded the required sample size of at least 27 individuals. All the baseline and follow-up variable data described above was stored in an Excel spreadsheet which was designed *ad hoc*. These metrics were: consumption of the AnMeD-S, physical and QoL characteristics at the beginning and end of the study; we also recorded whether there was an improvement for each of the characteristics for each individual participant. The advanced statistics software R was used for the statistical analyses. The qualitative variables were described as the sample sizes and percentages (*n* [%]); quantitative variables were described as the mean and standard deviation (x̄ ± s). To analyze the association between qualitative variables, Chi-squared or Fisher exact tests were used; statistical significance is indicated with an * if the *p-*value was <0.05; ** for <0.01, and *** for <0.001.

The difference in the scores achieved before and after the AnMeD-S intervention was used to determine the increase in consumption of the corresponding food group. Spearman rank correlations were calculated to evaluate if changes in consumption toward the diet recommendations given to participants were associated with the numerical indicators of QoL. Correlation tests and their corresponding *p*-values were also used to determine significant associations. In contrast with the methodology proposed in the previous paragraph, this procedure evaluated changes in these scores rather than dividing the individuals according to their adherence to the AnMeD-S consumption recommendations.

## 3. Results

### 3.1. Dietary bases for the nutritional intervention

The key information upon which the AnMeD-S anti-inflammatory nutritional trial conducted in this work was based was extracted from the academic literature. We designed the guide based on the MD according to the grade and accuracy of the available evidence. We chose the MD because it has been proven to reduce inflammation and oxidative stress and consequently, to increase cellular metabolism and promote stem cell-based regeneration. However, we also prohibited some foods included in the MD because they are considered pro-inflammatory (red meat, gluten, and cow’s milk), while also adding other anti-inflammatory foods such as curcumin and coffee.

Regarding the anti-inflammatory foods mentioned above, it is well known that polyphenols can effectively prevent and alleviate the symptoms of rheumatic diseases. This is because of their antioxidant and anti-inflammatory actions, which are mediated by inhibiting the transcription and expression of pro-inflammatory cytokines (35). Some important polyphenols are flavonols, anthocyanins, proanthocyanins, and resveratrol, which are present in many fruits and legumes. Moreover, carotenoids are known to have antioxidant properties because they inhibit reactive oxygen species (ROS), meaning they have a protective effect in rheumatic diseases. These carotenoids can be found in fruits and vegetables such as sweet potatoes, carrots, red peppers, squash, avocado, and watermelon.

At the same time, monounsaturated fatty acids (MUFAs), found in foods such as nuts, also have antioxidant activity which can mitigate chronic pro-inflammatory processes. Indeed, the function of polyunsaturated fatty acids (PUFAs) in the improvement of mitochondrial function, inhibition of inflammatory mediators such as interleukins (ILs) and tumor necrosis factor alpha (TNF-α), and decrease in oxidative stress is well-known. Importantly, there are different PUFAs known as ω-3 and ω-6, with both being involved in the inflammatory response and their ratio being of key importance. The ω-3 PUFAs are eicosapentaenoic acid (EPA) and docosahexaenoic acid (DHA), which both help in the regulation of antioxidant signaling pathways. In turn, the amount of the ω-6 PUFA, arachidonic acid (AA) released from cells determines the intensity of inflammation. When less AA is found in cell membranes, less AA is released and therefore, fewer eicosanoids are formed (36).

AA can mainly be found in the fatty parts of meats, especially red meat, while ω-3 is mainly found in EVOO, fish, and fish oil supplements. An increase in postprandial lipopolysaccharides (LPSs) and toll-like receptor-4 (TLR4) is associated with increased levels of inflammatory cytokines such as IL-6, IL-17, and TNF-α which, in turn, activate oxidative bursts. Many epidemiological studies have provided a solid rationale for the health benefits of diets based on foods of vegetable origin (36). Of note, an important aspect of vegetable consumption is mixing at least 2 or 3 vegetables with different colors in every meal to ensure the intake of different micronutrients. In this line, several studies have revealed that curcuminoids act to inhibit the expression of IL-1, IL-8, and IL-6, and TNF-α, thereby decreasing the severity of rheumatic disease. Moreover, these molecules also help to downregulate the production of NO, COX_2_, iNOS, lipoxygenase activator protein-1, and NF-kB in a wide range of cells, with the latter simultaneously increasing the levels of cytokines (IL-10).

Beta-casomorphin is a peptide with opioid activity which is produced by the incomplete degradation of the protein from cow’s milk (casein) in the intestine. Furthermore, increased intestinal permeability could lead to impairment of the intestinal barrier with the subsequent passage of such peptides into the bloodstream, thereby triggering a systemic immune response (37). Even though beta-casomorphin is mainly found in cow’s milk, consuming fermented foods such as plain yogurt and some cheeses has been shown to have a positive effect because of its impact on gut microbiota diversity (38). Thus, our nutritionist made the subjective decision of what foods to include in the AnMeD-S and the recommended frequency of their consumption, as shown in Table 2.

### 3.2. Characteristics of the study participants

Forty-five patients with an illness accompanied by CP were included in this study. Forty of the participants were women (89%) and the average age was 59 ± 7 years. Twenty patients had a background of cognitive impairment (44%), 40 patients claimed to have memory loss (89%), and possible cognitive impairment had been identified in 5 of them (11%). Finally, 28 patients had a diagnosis of depression (62%). The results from the cognitive impairment questionnaires were not statistically significant. This may have been because of our small sample size or because the participants were too young to be diagnosed. Moreover, it is important to note that following a new dietary routine for 4-months was insufficient to see any changes in the cognitive test results.

### 3.3. Design of the anti-inflammatory, supplemented, Mediterranean diet, and the assessment tools

Table 3 details the three main food groups. The first groups comprised foods that are part of a healthy diet. The second group defines foods with an anti-inflammatory effect. Finally, the last group contains foods with a pro-inflammatory effect. We also describe the consumption levels recommended for each food in order to achieve an anti-inflammatory therapeutic effect. To evaluate the diet, each food received a different score based on the recommended consumption frequency and its anti-inflammatory effect. We established a maximum score for each food, regardless of higher intake levels. A 100% consumption of healthy foods was graded with 60 points and a 100% consumption of anti-inflammatory foods was scored with a maximum of 96 points. Thus, the gold standard for this diet, if the patient followed the diet perfectly, was a total of 156 points.

**Table 3:** Recommended foods and their ideal consumption frequency with the score assigned to each food and the maximum score obtainable for each food group.

	Recommendations	Score	Score cap	Maximum score
Healthy diet foods				
White meat	3 servings of 100 g (approx.)/week	2 points/serving/week	6	60
White fish	3 servings of 125 g (approx.)/week	2 points/serving/week	6	
Legumes	3 servings of 80 g (approx.)/week	2 points/serving/week	6	
Eggs	Maximum of 4 eggs/week	−1 point for each additional egg/week	0	
Gluten free foods	1 serving of 50 g (approx.)/day	1 point/serving/day	7	
Greens	1 serving of 100 g (approx.)/day	1 point/serving/day	7	
Non-greens	1 serving of 100 g (approx.)/day	1 point/serving/day	7	
Other vegetables	1 serving of 100 g (approx.)/day	1 point/serving/day	7	
Enzymatic fruit	1 serving of 200 g (approx.)/day	1 point/serving/day	7	
Other fruit	1 serving of 200 g (approx.)/day	1 point/serving/day	7	
Increased anti-inflammatory foods				
Blue fish	3 servings of 125 g (approx.)/week	4 points/serving/week	12	96
Berries	1 serving of 100 g (approx.)/day	2 points/serving/day	14	
Extra virgin olive oil	3 tablespoons/day	2 points/3 tablespoons/day	14	
Nuts	1 serving of 30 g (approx.)/day	2 points/serving/day	14	
Plain yogurt or kefir	1 serving of 125 g (approx.)/day	2 points/serving/day	14	
Turmeric	4.5 g/day	3 points/serving/day	21	
Dark chocolate (>80% cocoa solids)	5 g/day	1 point/5 g/day	7	
Reduced pro-inflammatory foods				
Red meat	Not allowed	−1 point/serving/day	0	0
Refined grains	Not allowed	−1 point/serving/day	0	
Saturated fats	Not allowed	−1 point/serving/day	0	
Dairy products	Not allowed	−1 point/serving/day	0	
Coffee	Maximum of 2 cups/day	−1 point for each additional cup/day	0	
Alcohol	Not allowed	−1 point/serving/day	0	
Wine	Maximum of 1 glass/week	−1 point for each additional glass/week	0	
Sweetened beverages	Not allowed	−1 point/serving/day	0	
Sugar, pastries, and cake	Maximum of 5 g/day	−1 point/serving/day	0	
Sauces	Not allowed	−1 point/serving/day	0	
Total				156

### 3.4. Individual food intake and compliance with the nutritional recommendations

Table 4 shows the number of patients and their percentage adherence for each food, classified into four categories. Firstly, patients that already followed the recommendations and were able to maintain their habits (maintain column). Secondly, patients that did not follow the recommendations before starting the study but were able to meet them while completing the work (meet column). Thirdly, patients that improved their consumption habits without reaching the intake goal (improve column). Finally, patients that did not meet the required consumption recommendations or whose habits became worse (worsen column). It is worth noting that the consumption of healthy foods and anti-inflammatory foods is desirable, hence we considered an improvement to be an increase their consumption. Nevertheless, it is the other way around for pro-inflammatory foods, meaning that an improvement meant a decrease their consumption.

**Table 4:** The number of participants and percentages for their adherence to the anti-inflammatory, supplemented, Mediterranean diet.

	Worsen *n* (%)	Improve *n* (%)	Meet *n* (%)	Maintain *n* (%)
Healthy diet foods				
Poultry	2 (4)	2 (4)	11 (24)	30 (67)
White fish	9 (20)	6 (13)	13 (29)	17 (38)
Legumes	8 (18)	9 (20)	21 (47)	7 (16)
Eggs	7 (16)	0 (0)	4 (9)	34 (76)
Gluten-free foods	21 (47)	10 (22)	14 (31)	0 (0)
Greens	10 (22)	6 (13)	21 (47)	8 (18)
Non-greens	4 (9)	16 (36)	16 (36)	9 (20)
Other vegetables	22 (49)	11 (24)	9 (20)	3 (7)
Enzymatic fruit	23 (51)	10 (22)	4 (9)	8 (18)
Other fruit	20 (44)	9 (20)	10 (22)	6 (13)
Anti-inflammatory foods				
Blue fish	8 (18)	9 (20)	20 (44)	8 (18)
Berries	20 (44)	8 (18)	17 (38)	0 (0)
Extra virgin olive oil	4 (9)	0 (0)	8 (18)	33 (73)
Nuts	9 (20)	14 (31)	12 (27)	10 (22)
Plain yogurt or kefir	24 (53)	9 (20)	7 (16)	5 (11)
Turmeric	26 (58)	0 (0)	13 (29)	6 (13)
Dark chocolate (>80% cocoa solids)	27 (60)	8 (18)	3 (7)	7 (16)
Pro-inflammatory foods				
Red meat	15 (33)	0 (0)	19 (42)	11 (24)
Refined grains	18 (40)	0 (0)	24 (53)	3 (7)
Saturated fats	15 (33)	0 (0)	4 (9)	26 (58)
Milk and milk derivatives	7 (16)	0 (0)	23 (51)	15 (33)
Coffee	0 (0)	0 (0)	12 (27)	33 (73)
Alcohol	25 (56)	0 (0)	1 (2)	19 (42)
Wine	6 (13)	0 (0)	3 (7)	36 (80)
Sweetened beverages	3 (7)	0 (0)	6 (13)	36 (80)
Sugar, pastries, and cake	3 (7)	3 (7)	16 (36)	23 (51)
Sauces	7 (16)	1 (2)	18 (40)	19 (42)

From the beginning of the work, many of the patients were already eating some of the foods at the recommended frequencies. In the healthy foods group, 67 and 76% of the patients met this criteria for white meat and eggs, respectively. For the anti-inflammatory foods, 73% of the patients already met the EVOO consumption recommendation. Lastly, in the pro-inflammatory food category, 80% of the patients were already following the recommendations for wine and sweet beverages and 73% were following the coffee recommendation. In other words, the consumption of these foods was not responsible for any physical or QoL changes in this work because their consumption did not change during the course of the study. However, there was an improvement in the intake for several different foods including legumes (47%), greens (47%), non-greens (36%), blue fish (44%), and cow’s milk (51%). These improvements could have been involved in physical and QoL changes in the study participants. Furthermore, its seemed to be more complicated for participants to introduce certain foods into the diet such as gluten-free foods (47%), other vegetables (49%), enzymatic fruits (51%), plain yogurt or kefir (53%), and dark chocolate (60%), or for them to restrict refined grains (40%) and alcohol use (56%).

The number of participants and percentages that maintained adequate consumption according to our recommendations from the start of follow-up (Maintain), improved until complying with the recommendations (Meet), that improved but did not adhere to the recommendations (Improve), or whose consumption did not improve or worsened (Worsen).

In terms of foods associated with healthy diets, the consumption of poultry and eggs seemed to be easier for the participants to maintain. The patients met the established dietary requirements for legumes (47%) and greens (47%) and it was easier for them to improve their consumption of non-greens (36%), although not sufficiently to meet our recommendations. The anti-inflammatory food most easily maintained by almost all the patients was EVOO (73%). Blue fish was consumed in adequate levels in 44% of the participants and the ingestion of nuts was improved in only 31%.

### 3.5. Food consumption in anti-inflammatory, supplemented, Mediterranean diet during the intervention

For each food group, the participant consumption percentages were calculated at the beginning and end of the study. Patients were classified into three groups. The first group had the participants with a low consumption (0–50%); the second group comprised those with a moderate consumption (50–75%); and the last group was made up of patients with a high consumption (75–100%). Table 5 shows how the consumption levels contributed to each patient at the beginning (table rows) and end (table columns) of the study; it is divided into four groups: healthy foods, anti-inflammatory foods, pro-inflammatory foods, and overall diet. As we can observe, 27 patients consumed healthy foods sparingly; the consumption worsened to low consumption by the end of the study for 2 of participants; 11 patients maintained moderate consumption levels; and 14 patients improved to register high consumption.

**Table 5:** Distribution of the participants according to their consumption at the beginning and end of the follow-up in the different food groups and for the anti-inflammatory, supplemented, Mediterranean diet (AnMeD-S).

% of consumption before	Total (*n*)	% of consumption after		
		Low	Moderate	High
Healthy diet foods				
Low [0–50%]	15 (100; 33; 33)	4 (27; 67; 9)	10 (67; 45; 22)	1 (7; 6; 2)
Moderate [50–75%]	27 (100; 60; 60)	2 (7; 33; 4)	11 (41; 50; 24)	14 (52; 82; 31)
High [75–100%]	3 (100; 7; 7)	0 (0; 0; 0)	1 (33; 5; 2)	2 (67; 12; 4)
Total	45	6 (13; 100; 13)	22 (49; 100; 49)	17 (38; 100; 38)
Anti-inflammatory foods				
Low [0–50%]	29 (100; 64; 64)	11 (38; 73; 24)	13 (45; 81; 29)	5 (17; 36; 11)
Moderate [50–75%]	15 (100; 33; 33)	4 (27; 27; 9)	2 (13; 13; 4)	9 (60; 64; 20)
High [75–100%]	1 (100; 2; 2)	0 (0; 0; 0)	1 (100; 6; 2)	0 (0; 0; 0)
Total	45	15 (33; 100; 33)	16 (36; 100; 36)	14 (31; 100; 31)
Pro-inflammatory foods				
Low [0–50%]	28 (100; 62; 62)	26 (93; 68; 58)	2 (7; 40; 4)	0 (0; 0; 0)
Moderate [50–75%]	11 (100; 24; 24)	8 (73; 21; 18)	2 (18; 40; 4)	1 (9; 50; 2)
High [75–100%]	6 (100; 13; 13)	4 (67; 11; 9)	1 (17; 20; 2)	1 (17; 50; 2)
Total	45	38 (84; 100; 84)	5 (11; 100; 11)	2 (4; 100; 4)
Overall diet				
Low [0–50%]	37 (100; 82; 82)	13 (35; 87; 29)	15 (41; 87; 33)	9 (24; 75; 20)
Moderate [50–75%]	8 (100; 18; 18)	2 (25; 13; 4)	3 (38; 17; 7)	3 (38; 25; 7)
High [75–100%]	0 (100; 0; 0)	0 (0; 0; 0)	0 (0; 0; 0)	0 (0; 0; 0)
Total	45	15 (33; 100; 83)	18 (40; 100; 40)	12 (27; 100; 27)

The percentages after following the anti-inflammatory, supplemented, Mediterranean diet were classified as: Low [0–50%], n(% row, % col, % total), Moderate [50–75%], n(% row, % col, % total), and High [75–100%], n(% row, % col, % total). Red identifies patients whose consumption worsened in any group, amber was used for patients who maintained their consumption, and green was used for patients whose consumption improved.

If we compare the evolution of the patients, we can see that 38 of them improved their consumption of pro-inflammatory foods compared to 29 patients in the anti-inflammatory foods group and 27 in the healthy diet group. This information indicates that, *a priori* it is easier for patients to abandon unhealthy habits in their diet than to incorporate healthy habits. On the one hand, healthy and anti-inflammatory foods were assessed positively; therefore, to achieve a good score for the AnMeD-S it was advisable to consume high amounts of them. On the other hand, pro-inflammatory foods were assessed negatively and hence, it was advisable to consume the lowest amount of them possible. In accordance with this scenario, to easily pinpoint each patient in each group that had improved or worsened, in Table 5 we highlighted them in green or red, respectively; the amber color was used only in the healthy food group for patients who had maintained their consumption. For instance, the consumption of healthy foods worsened in 7 patients, stayed moderate in 11, and improved in 27.

### 3.6. Association between food consumption and physical improvement

Physical characteristics and the improvement or worsening of the patient metrics recorded are shown in the rows in Table 6. An improvement was considered a decrease in the variable, except for the MNA, corporal water content, and muscular mass. In turn, the columns represent an improvement or worsening in each food group. As discussed, changes in the consumption of healthy foods were not statistically associated with the physical condition of the participants. Nevertheless, a reduction in the consumption of anti-inflammatory foods was positively associated with a decrease in BMI, BF, and AC and WC. Likewise, improvements in pro-inflammatory food consumption was positively associated with decreased weight, BMI, BF, MA, VF, and WC and HC.

**Table 6:** Association between improved food intake and improved physical characteristics.

Physical enhancement		Healthy diet foods				Anti-inflammatory foods			Pro-inflammatory foods			Overall diet		
	Totals	Worse 7 (100%)	No change 11 (100%)	Improve 27 (100%)	*p*-value	Worse 16 (100%)	Improve or no change 29 (100%)	*p*-value	Worse or no change 6 (100%)	Improve 39 (100%)	*p*-value	Worse 15 (100%)	Improve or no change 30 (100%)	*p*-value
MNA^¥^														
Decrease	24 (53)	2 (29)	8 (73)	14 (52)	0.182^a^	7 (44)	17 (59)	0.369^a^	4 (67)	20 (51)	0.670^a^	8 (53)	16 (53)	1.000^a^
Increase	21 (47)	5 (71)	3 (27)	13 (48)		9 (56)	12 (41)		2 (33)	19 (49)		7 (47)	14 (47)	
Weight														
Increase	16 (36)	3 (43)	5 (46)	8 (30)		9 (56)	7 (24)		5 (83)	11 (28)		8 (53)	8 (27)	
Decrease	29 (64)	4 (57)	6 (55)	19 (70)	0.592^a^	7 (44)	22 (76)	0.051^a^	1 (17)	28 (72)	**0.017**^b^*	7 (47)	22 (73)	0.105^a^
BMI														
Increase	18 (40)	3 (43)	5 (46)	10 (37)	0.879^a^	11 (69)	7 (24)	**0.005**^a^**	6 (100)	12 (31)	**0.002**^b^**	10 (67)	8 (27)	**0.022**^a^*
Decrease	27 (60)	4 (57)	6 (55)	17 (63)		5 (3)	22 (76)		0 (0)	27 (69)		5 (33)	22 (73)	
BF														
Increase	7 (16)	1 (14)	3 (27)	3 (11)		5 (31)	2 (7)		4 (67)	3 (8)		6 (40)	1 (3)	
Decrease	38 (84)	6 (86)	8 (73)	24 (89)	0.457^b^	11 (69)	27 (93)	0.079^a^	2 (33)	36 (92)	**0.003**^a^**	9 (60)	29 (97)	**0.003**^b^**
BW														
Decrease	7 (16)	2 (29)	3 (27)	2 (7)		4 (25)	3 (10)		3 (50)	4 (10)		6 (40)	1 (3)	
Increase	38 (84)	5 (71)	8 (73)	25 (93)	0.181^a^	12 (75)	26 (90)	0.225^a^	3 (50)	35 (90)	**0.039**^a^*	9 (60)	29 (97)	**0.003**^b^**
MM														
Decrease Increase	10 (22) 35 (78)	2 (29) 5 (71)	3 (27) 8 (73)	5 (19) 22 (82)	0.763^a^	5 (31) 11 (69)	5 (17) 24 (83)	0.455^a^	2 (33) 4 (67)	8 (21) 31 (80)	0.601^a^	7 (47) 8 (53)	3 (10) 27 (90)	**0.009**^a^**
MA														
Increase Decrease	25 (56) 20 (44)	1 (14) 6 (86)	6 (55) 5 (46)	18 (67) 9 (33)	0.045^b^	10 (63) 6 (38)	15 (52) 14 (48)	0.544^a^	6 (100) 0 (0)	19 (49) 20 (51)	**0.027**^b^*	9 (60) 6 (40)	16 (53) 14 (47)	0.757^a^
VF														
Increase Decrease	18 (40) 27 (60)	2 (29) 5 (71)	5 (46) 6 (55)	11 (41) 16 (59)	0.770^a^	11 (69) 5 (3)	7 (24) 22 (76)	**0.005**^a^**	6 (100) 0 (0)	12 (31) 27 (69)	**0.002**^b^**	10 (67) 5 (33)	8 (27) 22 (73)	**0.022**^a^*
AP														
Increase Decrease	21 (47) 24 (53)	3 (43) 4 (57)	6 (55) 5 (46)	12 (44) 15 (56)	0.832^a^	12 (75) 4 (25)	9 (31) 20 (69)	**0.006**^a^**	4 (67) 2 (33)	17 (44) 22 (56)	0.396^a^	11 (73) 4 (27)	10 (33) 20 (67)	**0.025**^a^*
WP														
Increase Decrease	17 (38) 28 (62)	5 (71) 2 (29)	4 (36) 7 (64)	8 (30) 19 (70)	0.126^a^	10 (63) 6 (38)	7 (24) 22 (76)	**0.023**^a^*	5 (83) 1 (17)	12 (31) 27 (69)	**0.023**^b^*	10 (67) 5 (33)	7 (23) 23 (77)	**0.008**^a^**
HP														
Increase Decrease	16 (36) 29 (64)	3 (43) 4 (57)	2 (18) 9 (82)	11 (41) 16 (59)	0.381^a^	9 (56) 7 (44)	7 (24) 22 (76)	0.051^a^	5 (83) 1 (17)	11 (28) 28 (72)	**0.017**^b^*	9 (60) 6 (40)	7 (23) 23 (77)	**0.023**^a^*
WHI														
Increase Decrease	25 (56) 20 (44)	6 (86) 1 (14)	7 (64) 4 (36)	12 (44) 15 (56)	0.121^b^	12 (75) 4 (25)	13 (45) 16 (55)	0.066^a^	3 (50) 3 (50)	22 (56) 17 (44)	1.000^a^	12 (80) 3 (20)	13 (43) 17 (57)	**0.027**^a^*

^a^ Chi-squared test. ^b^Fisher exact test. *p < 0.05. **p < 0.01. ^¥^MNA was classified as a physical enhancement because it measures changes in nutritional status; it was not considered a QoL metric. Values in bold represent statistically significant associations.

Finally, on the one hand, the overall diet was positively associated with a decreased BMI, BF, VF, AC, WC, HC, and WHI. It was also associated with an increase in BW and MM. On the other hand, having chronic pain disease was not associated with any improvements in physical characteristics. Table 7 shows the anthropometric measurements recorded for the participants before and after the nutritional intervention.

**Table 7:** Physical characteristics before and after the anti-inflammatory, supplemented, Mediterranean diet.

Physical characteristics	Before AnMeD-S	After AnMeD-S
MNA^¥^	11.6 ± 1.9	12.2 ± 1.6
Malnourished [0.7]	0 (0)	0 (0)
Malnutrition risk [8, 12]	15 (33)	13 (29)
Well-nourished [12, +]	30 (67)	32 (81)
Weight	71.9 ± 13.2	71.4 ± 13.2
BMI	27.2 ± 5.0	26.8 ± 4.8
Underweight [0, 18.5]	1 (2)	1 (2)
Normal [18.5, 25]	17 (38)	16 (36)
Overweight [25, 30]	17 (38)	16 (36)
Obese [30, +]	10 (22)	12 (27)
BF	37.0 ± 8.5	32.0 ± 7.8
Bass	0 (0)	2 (4)
Healthy	20 (44)	28 (62)
Tall	9 (20)	8 (18)
Obese	16 (36)	7 (16)
BW	45.7 ± 6.2	50.3 ± 5.1
Insufficient	20 (44)	5 (11)
Healthy	25 (56)	40 (89)
MM	42.2 ± 7.1	44.9 ± 6.6
MA	44.2 ± 26.5	53.6 ± 12.5
VF	8.1 ± 6.7	9.0 ± 4.2
Healthy (1–12)	40 (89)	38 (84)
Excess (13–59)	5 (11)	7 (16)
AC	30.0 ± 3.8	28.8 ± 3.7
WC	93.5 ± 14	92.4 ± 20.4
HC	106.3 ± 12.8	104.0 ± 12.3
WHI	0.9 ± 0.1	0.9 ± 0.2
Normal	24 (53)	29 (64)
High	21 (47)	16 (36)

The quantitative variables were summarized as the mean plus or minus the standard deviation ( $\bar{x\pm sd}$ ) and qualitative variables were summarized as the sample size and percentage (n [%]). ^¥^MNA was classified as a physical enhancement because it measures changes in nutritional status; it was not considered a QoL metric. AnMeD-S, anti-inflammatory, supplemented, Mediterranean diet; MNA, Mini Nutritional Assessment; BMI, weight, body mass index; BF, body fat; BW, body water; MM, muscle mass; MA, metabolic age; VF, visceral fat; AC, arm circumference; WC, waist circumference; HC, hip circumference; WHI, waist-hip index.

### 3.7. Association between food intake and improvement in quality-of-life attributes

Table 8 shows the changes in QoL characteristics among our participants. The patients improved when the value for each characteristic had decreased at the end of the study, with the exception of rest and sleep satisfaction in which an improvement was an increase in the value. The columns in the table indicate the changes for each food group and for the overall diet. No statistically significant associations were observed between an increase in the consumption of healthy foods and improvement in any of the QoL characteristics. However, equal, or greater consumption of anti-inflammatory foods was associated with decreased pain and stress. In addition, reduced consumption of pro-inflammatory foods was associated with positive changes in sleep satisfaction. At the end of the study, an improvement in the overall diet was positively correlated a decrease in pain, HAQ score, and hypersomnia.

**Table 8:** Association between food intake and quality of life metrics.

Quality of life characteristics		Healthy diet foods				Anti-inflammatory foods			Pro-inflammatory foods			Overall diet		
	Totals	Worse 7 (100%)	No change 11 (100%)	Improve 27 (100%)	*p*-value	Worse 16 (100%)	Improve or no change 29 (100%)	*p*-value	Worse or no change 6 (100%)	Improve 39 (100%)	*p*-value	Worse 15 (100%)	Improve or no change 30 (100%)	*p*-value
Pain														
Increase Decrease	15 (33) 30 (67)	2 (29) 5 (71)	6 (55) 5 (45)	7 (26) 20 (74)	0.227^a^	9 (56) 7 (44)	6 (21) 23 (79)	**0.02**3^a^*	3 (50) 3 (50)	12 (31) 27 (69)	0.384^a^	10 (67) 5 (33)	5 (17) 25 (83)	**0.002**^a^**
Stress														
Increase Decrease	20 (44) 25 (56)	2 (29) 5 (71)	7 (64) 4 (36)	11 (41) 16 (59)	0.286^a^	11 (69) 5 (31)	9 (31) 20 (69)	**0.027**^a^*	4 (66) 2 (33)	16 (41) 23 (59)	0.383^a^	10 (67) 5 (33)	10 (33) 20 (67)	0.056^a^
Depression														
Increase Decrease	14 (31) 31 (69)	3 (43) 4 (57)	5 (46) 6 (55)	6 (22) 21 (78)	0.286^a^	5 (31) 11 (69)	9 (31) 20 (69)	1.000^a^	2 (33) 4 (67)	12 (31) 27 (69)	1.000^a^	5 (33) 10 (67)	9 (30) 21 (70)	1.000^a^
Disability HAQ														
Increase Decrease	24 (53) 21 (47)	4 (57) 3 (43)	9 (82) 2 (18)	11 (41) 16 (59)	0.069^a^	11 (69) 5 (31)	13 (45) 16 (55)	0.212^a^	4 (67) 2 (33)	20 (51) 19 (49)	0.670^a^	12 (80) 3 (20)	12 (40) 18 (60)	**0.014**^a^*
Rest														
Decrease Increase	18 (40) 27 (60)	2 (29) 5 (71)	5 (46) 6 (55)	11 (41) 16 (59)	0.770^a^	4 (25) 12 (75)	14 (48) 15 (52)	0.204^a^	2 (33) 4 (67)	16 (41) 23 (59)	1.000^a^	4 (27) 11 (73)	14 (47) 16 (53)	0.333^a^
Sleep satisfaction														
Decrease Increase	25 (56) 20 (44)	4 (57) 3 (43)	5 (45) 6 (55)	16 (59) 11 (41)	0.737^a^	11 (69) 5 (31)	14 (48) 15 (52)	0.224^a^	6 (100) 0 (0)	19 (49) 20 (51)	**0.027**^b^*	10 (67) 5 (33)	15 (50) 15 (50)	0.352^a^
Hypersomnia														
Increase Decrease	28 (62) 17 (38)	3 (43) 4 (57)	9 (82) 2 (18)	16 (59) 11 (41)	0.222^a^	12 (75) 4 (25)	16 (55) 13 (45)	0.219^a^	5 (83) 1 (17)	23 (59) 16 (41)	0.385	13 (87) 2 (13)	15 (50) 15 (50)	**0.023**^a^*
Insomnia														
Increase Decrease	13 (29) 32 (71)	1 (14) 6 (86)	4 (36) 7 (64)	8 (30) 19 (70)	0.597^b^	7 (44) 9 (56)	6 (21) 23 (79)	0.169^a^	4 (67) 2 (33)	9 (23) 30 (77)	0.049	7 (47) 8 (53)	6 (20) 24 (80)	0.086^a^

^a^Chi-squared test. ^b^Fisher exact test. *p < 0.05, **p < 0.01. Values in bold represent statistically significant associations.

**Figure 2:**
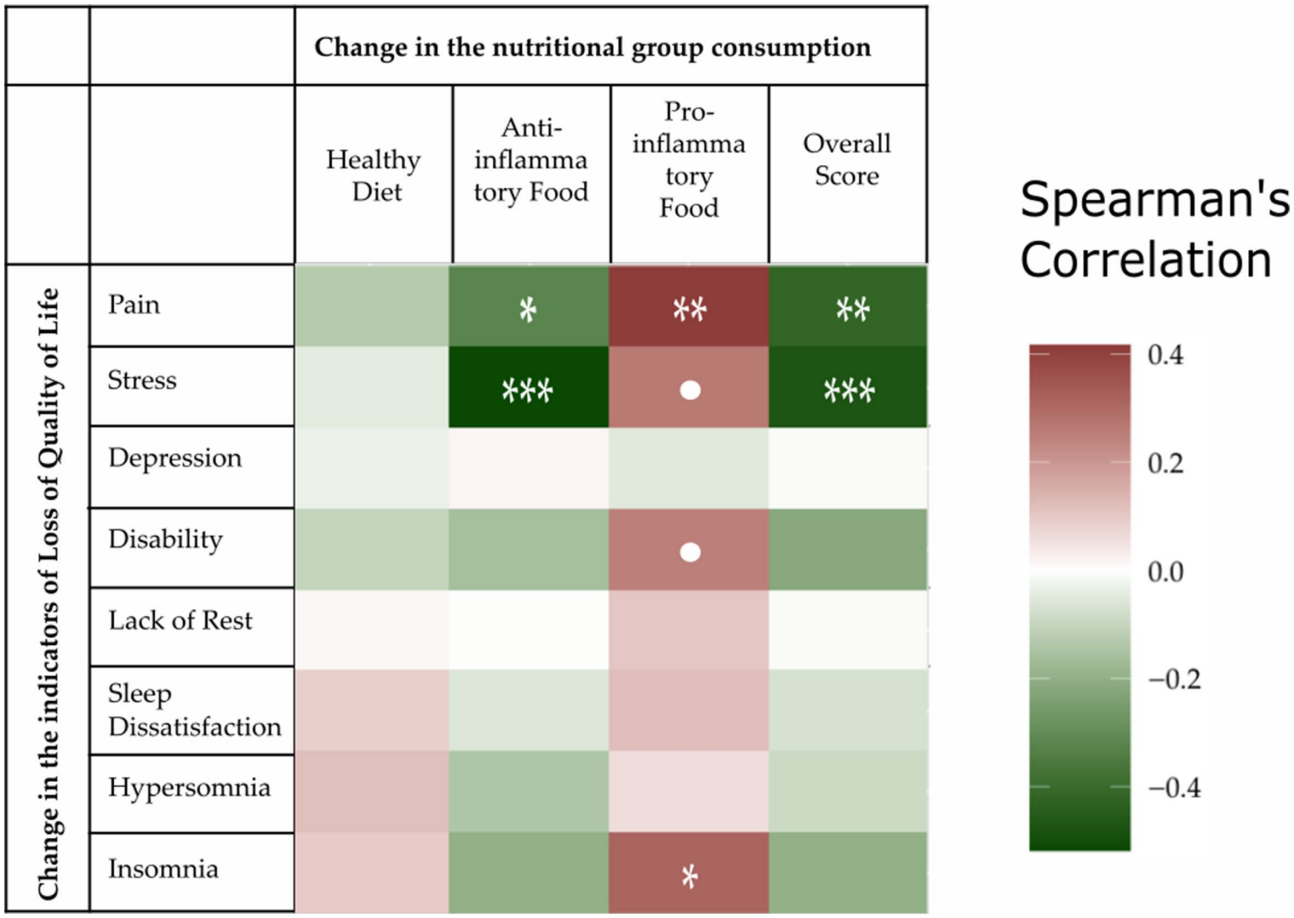
Spearman rank correlations of the change in the nutritional score and quality of life (QoL) indicators. All the scores were scaled so that a deterioration in QoL implied an increased value. The “pain,” “stress,” and “lack of rest” characteristics were measured with a numerical rating scale; “depression” was measured with the Geriatric Depression Scale (GDS); “disability” with the Health Assessment Questionnaire (HAQ); and “insomnia,” “hypersomnia,” and “dissatisfaction” corresponded to the same items in the Oviedo Sleep Questionnaire (OSQ). Specifically, we considered sleep dissatisfaction and lack of rest rather than satisfaction and rest. Negative correlation values for food/indicator pairs were shaded in green and represent improvements in patient QoL. Conversely, positive correlation values were shaded in red and represented a deterioration in the quality of life. •*p* < 0.1, *p < 0.05, **p < 0.01, and ***p < 0.001.

To analyze data from a different perspective, we calculated Spearman rank correlations for the changes in nutritional scores and the scales measuring QoL characteristics. Firstly, these tests did not indicate any association between QoL characteristics and the general consumption of a healthy diet. Secondly, the correlations suggested an association between pain and the anti-inflammatory score (Spearman’s rho = −0.32, *p* = 0.0315), pain and the pro-inflammatory score (Spearman’s rho = −0.42, *p* = 0.0045), pain and the overall score (Spearman’s rho = −0.42, *p* = 0.0043), stress and an anti-inflammatory diet (Spearman’s rho = −0.51, *p* = 0.0003), and stress and the overall score (Spearman’s rho = −0.48, *p* = 0.0010). Figure 2 graphically represents the Spearman correlations for all the score/QoL characteristic pairs as a grid, with a higher correlation intensity with an improvement in QoL shown in green. The numerical values of the Spearman correlations and outcomes of each test are provided in Supplementary Table S1.

**Figure 3:**
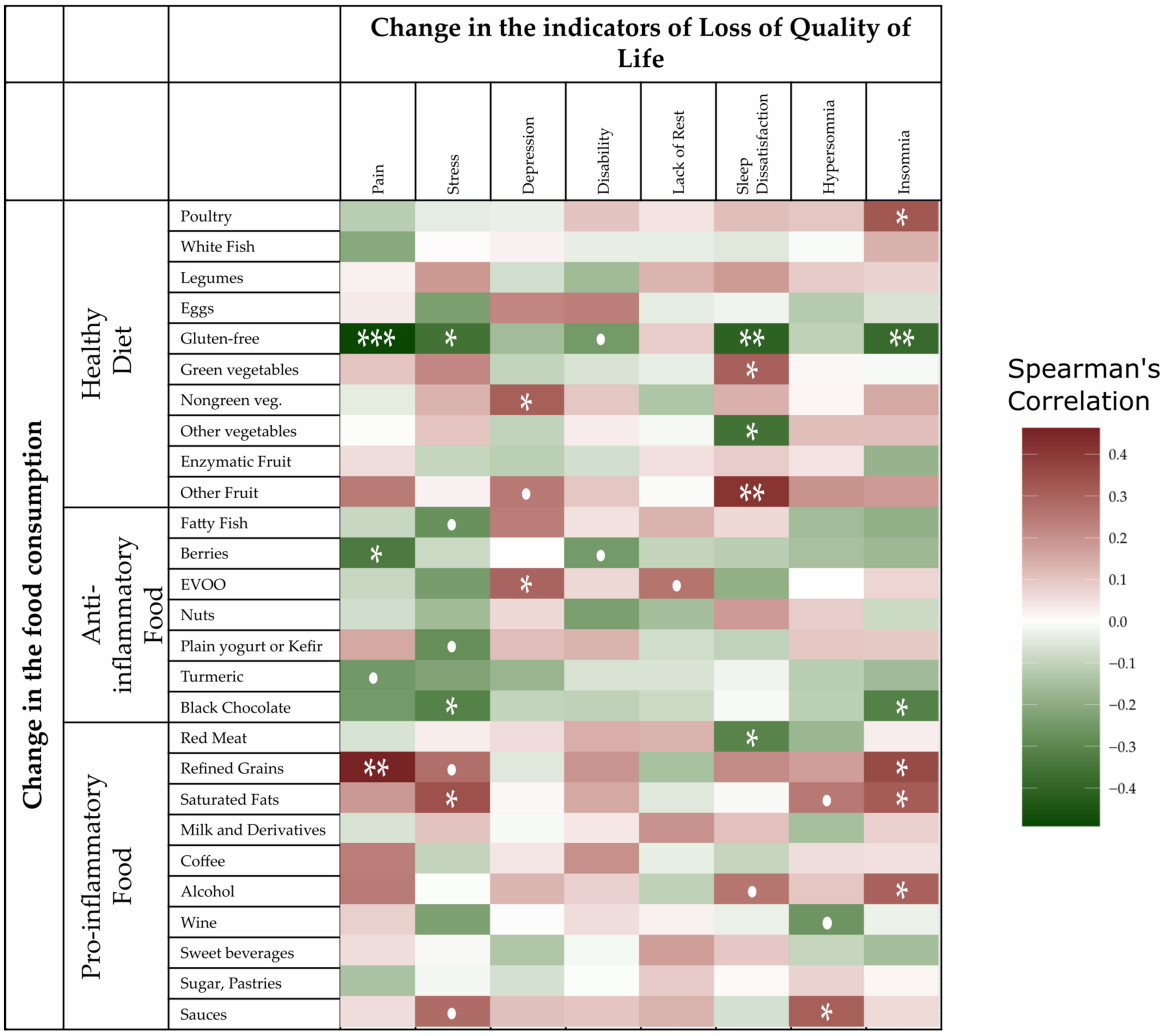
Spearman rank correlations of the change in the consumption of foods according to the nutritional score and changes in the quality of life (QoL) indicators. All the scores were scaled so that a deterioration in QoL implied an increased value. The “pain,” “stress,” and “lack of rest” characteristics were measured with a numerical rating scale; “depression” was measured with the Geriatric Depression Scale (GDS); “disability” with the Health Assessment Questionnaire (HAQ); and “insomnia,” “hypersomnia,” and “dissatisfaction” corresponded to the same items in the Oviedo Sleep Questionnaire (OSQ). Specifically, we considered sleep dissatisfaction and lack of rest rather than satisfaction and rest. Negative correlation values for food/indicator pairs were shaded in green and represent improvements in patient QoL. Conversely, positive correlation values were shaded in red and represented a deterioration in the quality of life. •*p* < 0.1, *p < 0.05, **p < 0.01, and ***p < 0.001.

The Spearman rank correlation between food groups and QoL characteristics was not presented in Figure 3. These correlation tests indicated some associations between pain and food groups, especially with gluten-free foods (Spearman’s rho = −0.49, *p* = 0.0006), refined grains (Spearman’s rho = 0.46, *p* = 0.0014), and berries (Spearman’s rho = −0.3341, *p* = 0.02492). The correlations for the other groups were not so clear and so were graphically represented with a heatmap in Figure 3; their Spearman rank correlation values and *p*-values are shown in Supplementary Table S2. In Figure 2, higher correlations with a deterioration in QoL were shown as a higher intensity red color, while higher intensity green shows a greater improvement in the QoL characteristics. A diagnosis of chronic disease was not associated with improvements in QoL attributes. Of note, even though we found a significant positive association between improved food consumption and better QoL metrics, this does not inherently imply a cause–effect relationship.

### 3.8. Association between pain improvement and improvements in quality-of-life metrics

Pain can reduce or condition well-being in the daily lives of patients. We analyzed the association between pain and changes in their everyday anthropometric characteristics. As shown in Table 9, decreased pain was positively associated with improved stress, HAQ, hypersomnia, and insomnia.

**Table 9:** Associations between improvement in pain and quality of life characteristics.

Quality of life characteristics	Totals	Pain		
		Increase 15 (100)	Decrease 30 (100)	*p*-value
Stress				
Increase Decrease	20 (44) 25 (56)	11 (73) 4 (27)	9 (30) 21 (70)	**0.010**^a^*
Depression				
Increase Decrease	14 (31) 31 (69)	6 (40) 9 (60)	8 (27) 22 (73)	0.497^a^
Disability HAQ				
Increase Decrease	24 (53) 21 (47)	12 (80) 3 (20)	12 (40) 18 (60)	**0.014**^a^*
Rest				
Decrease Increase	18 (40) 27 (60)	7 (47) 8 (53)	11 (37) 19 (63)	0.538^a^
Sleep satisfaction				
Decrease Increase	25 (56) 20 (44)	11 (73) 4 (27)	14 (47) 16 (53)	0.118^a^
Hypersomnia				
Increase Decrease	28 (62) 17 (38)	14 (93) 1 (7)	14 (47) 16 (53)	**0.003**^b^**
Insomnia				
Increase Decrease	13 (29) 32 (71)	10 (67) 5 (33)	3 (10) 27 (90)	**<0.001**^a^***

^a^Chi-squared test. ^b^Fisher’s exact test. *p < 0.05, **p < 0.01, ***p < 0.001. Values in bold represent statistically significant associations.

HAQ, Health Assessment Questionnaire.

## 4. Discussion

Given the extension of western eating patterns in our culture (47), and the increasing presence of inflammation-based diseases (with or without an identifiable clinical origin) with CP (48), we set out to conduct a study to investigate the effect of an anti-inflammatory diet in patients with diagnoses associated with CP. Similar to studies examining the prevalence of RA, OA, and FM (49), there were more women in our study sample (88.9%) than men. Indeed, most published studies include mainly female patients because the prevalence of rheumatic disease is higher in women (92%) than in men (3, 49). Therefore, in this sense, our results could be considered representative of the general population.

We had two main goals in this research. First, we performed a systematic literature review to compile updated knowledge on the topic and to prepare a nutritional guide (the AnMeD-S) for patients with CP that included anti-inflammatory foods. Second, after fulfilling this first objective, we conducted an intervention to evaluate the efficacy of this guide and to analyze the benefits of consuming this diet in terms of physical parameters (weight, BMI, BF, BW, MM, MA, VF, AC, WC, HC, and WHI) and QoL characteristics (pain, stress, depression, disability, sleep quality, and cognitive impairment).

Numerous previous studies (4, 24, 50–52) have established the benefits of such diets in CP associated with different pathologies. Of note, within rheumatic diseases, there are differences regarding the origin of the pain, with RA being caused by chronic inflammation processes while, for example, FM seems to be related to alterations in the central modulation of pain. In our study, the result of the intervention was homogeneous regardless of the original diagnosis. Therefore, the AnMeD-S could also be recommended for other pathologies that present with CP. A recent systematic review and meta-analysis conducted by Wu et al. (53) focused, for the first time, on the relationship between the MD and inflammatory processes, finding an inverse association between them. The AnMeD-S includes a series of modifications to the MD, although it maintains recommendations for the consumption of foods considered healthy and it also promotes the consumption of anti-inflammatory foods and forbids the consumption of those considered pro-inflammatory.

The strategy of modifying or restricting some food types in rheumatic diseases has been evaluated by different authors with varying success. Rodrigo et al. (54) evaluated a gluten-free diet in patients with FM and, using a visual analog scale (VAS) for pain, observed a notable improvement this parameter. In our nutritional intervention with the AnMeD-S, we limited dairy consumption to one plain yogurt/kefir a day, eliminated wine, restricted gluten-containing foods, and included turmeric as a supplement. Notably, after a reduction in foods with gluten, we observed significant improvements in the QoL indicators of pain, stress, sleep dissatisfaction, and insomnia. Pain also seemed to improve with the consumption of berries and turmeric, while eating dark chocolate improved stress and insomnia.

Marum et al. (55) administered a low fermentable oligosaccharides, disaccharides, monosaccharides, and polyols (FODMAP) diet to patients with FM for 4 months and observed a statistically significant reduction in the mean VAS scores for pain after the intervention. In our case, we did not restrict fruits or vegetables, which can be sources of FODMAPs. However, the participants were advised not to consume dairy products as sources of lactose (they were only allowed to consume one plain yogurt or kefir per day), or sugary drinks as a source of high fructose corn syrup. There was an improvement in pain in both cases, although this did not reach statistical significance.

There are few published nutritional intervention studies in patients with OA, and those that are available were fundamentally aimed at weight loss because this is one of the recommendations in the guidelines for rheumatic diseases. However, the meta-analyses conducted by Smedslund et al. (56), did not show any consistent effect on pain for these dietary interventions. In addition, numerous studies have analyzed different foods and/or diets in the development and evolution of RA. For example, Johansson et al. (57) found that greater adherence to the MD was related to a lower risk of suffering RA. Although the MD varies from one country to another, it always involves the high consumption of monounsaturated fatty acids (vegetables, fruits, legumes, nuts, and fish) and moderate consumption of dairy products, meat, and wine. Different studies on the MD have shown that it is related to improvements in inflammation markers. Thus, increasing the consumption of fish, long-chain ω-3 PUFAs, and tea lowered the risk of suffering from RA (58–60). However, it was impossible to relate this decreased inflammation to the consumption of certain foods, although MUFAs (legumes, nuts, vegetables, and fruits) seem to be essential.

Regarding the reduction in pain and other QoL parameters, various studies have observed that MD has a greater effect on pain than vegetarian or vegan diets. However, it must be considered that, in general, most patients with rheumatism generally have poor quality diets and fail to meet the daily requirements for many nutrients. Thus, modifying these consumption habits to approximate them to a MD diet improves the QoL of these patients (3, 61, 62). In our study we analyzed the habitual consumption (and portions used) of the foods included in each of the 4 defined groups, both before and after the intervention, assigning each patient the corresponding score. Patients were classified as those with low consumption (0–50%), moderate consumption (50–75%), or high consumption (75–100%). We observed that the starting point was a high percentage of patients who consumed the healthy diet and anti-inflammatory foods but also consumed pro-inflammatory foods. However, by the end of the study, only two patients maintained high or moderate consumption of pro-inflammatory foods. In general, their physical characteristics improved after the nutritional intervention, although there were no statistically significant differences. However, we measured statistically significant differences in terms of improved pain and stress when analyzing the QoL parameters.

It was especially noteworthy that most participants consumed foods in line with our recommendations. Interestingly, the group of healthy foods consumed included legumes, greens, and non-greens, while in the group of anti-inflammatory foods, the ingestion of blue fish increased. It is also worth highlighting that these foods coincided with those identified by other authors (31, 62) as being anti-inflammatory and are present in almost every proposed anti-inflammatory diet. Hence, this result predicts, *a priori*, the effectiveness of the AnMeD-S because the inclusion of these foods did not seem to be difficult for the participants in our cohort. In addition, most of the patients limited the consumption of foods such as refined cereals, milk, and milk derivatives to comply with the recommended diet. In contrast, other foods (dark chocolate, alcohol, plain yogurt or kefir, enzymatic fruits, and turmeric) were consumed in the opposite way to our recommendations. These results are especially interesting because they may represent sticking points that will be relevant in future education. New proposals for anti-inflammatory diets should recommend generalizing or regulating the consumption of foods such as kefir, turmeric, and dark chocolate, as is already the case for alcohol.

When we compared patients who only improved their diet to those whose consumption came close to meeting our recommendations, we saw that for our cohort it was easier to comply with the recommendations than just to try to do so, especially for the pro-inflammatory food group. This highlights the efficacy of interventions to truly incorporate healthy lifestyle habits. In our work, the withdrawal of pro-inflammatory foods was significantly associated with several variables linked to the improvement of the physical characteristics of patients (specifically, weight, BMI, MA, VF, WC, and HC). Moreover, the consumption of anti-inflammatory foods was also significantly associated with improvements in some physical characteristics (AC, HC, VF, and BMI). This finding suggests that controlling pro-inflammatory foods will be important in interventions that aim to have a greater effect on physical parameters. Rapid implementation by patients and a clear effect on the physical parameters make the control of pro-inflammatory foods particularly appropriate in pathologies such as cardiovascular disease which require early intervention. In fact, several studies have already shown that a better health prognosis (63) is associated with improved physical variables for cardiometabolic risk (64), such as those recorded in this current work (weight and BMI, etc.).

Different studies have evaluated the consumption of nutritional supplements with possible anti-inflammatory or antioxidant properties. The Mathieu et al. meta-analysis (65) reported that curcumin and ginger supplementation could favorably impact knee OA symptoms, including pain reduction, but found no clear conclusions when supplementing with omega-3 or vitamin D. However, another meta-analysis which considered every type of rheumatic disease found a positive correlation between PUFA supplementation, especially omega-3 from animal sources (>2 g/day), and an improvement of the symptoms of these diseases (66). Of note, the traditional medicine of certain cultures, especially in Asia, uses spices such as garlic, ginger, cinnamon, and saffron to for rheumatic diseases because of their anti-inflammatory effects. In this sense, different studies have shown improvements in these diseases when using a combination of spices both in terms of subjective measures (VAS pain) and objective metrics (C-reactive protein) (67).

However, perhaps turmeric has been most widely used in anti-inflammatory treatments in traditional Chinese and Ayurvedic medicines. The rhizome of the *Curcuma longa* plant is used as the main active ingredient because its essential oil and curcuminoids (of which the most important is curcumin) have beneficial properties for health. Indeed, various studies have shown that turmeric consumption improved the severity of inflammation and reduced pain in patients with rheumatic diseases (68). In our study, patients were provided with a container of turmeric (*Curcuma Latte*) that they had to consume throughout the intervention. In this current study, this product was shown to significantly reduce pain and improve stress and depression, although patients did not enjoy its flavor and found it difficult to consume.

### 4.1. Study limitations and strengths

The limitations of the study were that the follow-up time was only 4 months and so it was impossible to know if the patients maintained their modified eating habits once the intervention period had finished and the difficulty in obtaining a routine assessment of blood chemistry parameters and inflammation markers of the participants. However, the modifications and restrictions in the AnMeD-S were relatively easy for patients to apply and follow, which should have helped ensure that they did not easily abandon it.

## 5. Conclusion

To conclude, this present study provided dietary guidelines which emphasized the consumption of anti-inflammatory foods known to be related to the relief of CP and to improve stress, depression, and sleep disturbances. The homogeneity of our study sample should help the extrapolation of the findings to other populations with CP. Nevertheless, we used a small sample size in this pilot study, meaning that further studies with larger sample cohorts will be required to corroborate the relationships we identified and to confirm the effectiveness of the AnMeD-S in groups of patients suffering from pain associated with inflammation.

## Data availability statement

The raw data supporting the conclusions of this article will be made available by the authors, without undue reservation.

## Ethics statement

The studies involving human participants were reviewed and approved by the Institutional Review Board (IRB) at the CEU Cardenal Herrera University (CEEI20/095, approval date: 31 March 2021). The patients/participants provided their written informed consent to participate in this study.

## Author contributions

LM, MD-A, MS-C, and TLC: conceptualization, methodology, writing original draft preparation, and writing review and editing. FM and MA: software and formal analysis. MS-C and TLC: investigation. MG and ML-R: data curation and review. LM: funding acquisition. All authors contributed to the article and approved the submitted version.

## Funding

This research was funded by the Cathedra DeCo MICOF-UCH University, the Regional Ministry of Innovation, Universities, Science, and the Digital Society of the Valencian Community (GV/2021/002), and SANTANDER-CEU (FUSP-BS-PPC26/2018). TLC was supported by the Research Fellowship grant from “Ayudas a la Formación de Jóvenes Investigadores CEU-Santander.”

## Conflict of interest

The authors declare that the research was conducted in the absence of any commercial or financial relationships that could be construed as a potential conflict of interest.

## Publisher’s note

All claims expressed in this article are solely those of the authors and do not necessarily represent those of their affiliated organizations, or those of the publisher, the editors and the reviewers. Any product that may be evaluated in this article, or claim that may be made by its manufacturer, is not guaranteed or endorsed by the publisher.

## References

[1] Kosek E Cohen M Baron R Gebhart GF Mico JA Rice ASC. Do we need a third mechanistic descriptor for chronic pain states? Pain. (2016) 157:1382–6. doi: 10.1097/j.pain.0000000000000507 26835783

[2] World Heart Federation. (2021). Available at: https://world-heart-federation.org

[3] Schönenberger KA Schüpfer AC Gloy VL Hasler P Stanga Z Kaegi-Braun N. Effect of anti-inflammatory diets on pain in rheumatoid arthritis: a systematic review and meta-analysis. Nutrients. (2021) 13:4221. doi: 10.3390/nu13124221 34959772 PMC8706441

[4] Häuser W Fitzcharles MA. Facts and myths pertaining to fibromyalgia. Dialogues Clin Neurosci. (2018) 20:53–62. doi: 10.31887/DCNS.2018.20.1/whauser, PMID: 29946212 PMC6016048

[5] Elma Ö Yilmaz ST Deliens T Coppieters I Clarys P Nijs J. Do nutritional factors interact with chronic musculoskeletal pain? A systematic review. J Clin Med. (2020) 9:702. doi: 10.3390/jcm9030702, PMID: 32150934 PMC7141322

[6] Duque L Fricchione G. Fibromyalgia and its new lessons for neuropsychiatry. Med Sci Monit Basic Res. (2019) 25:169–78. doi: 10.12659/MSMBR.915962, PMID: 31273184 PMC6636402

[7] Bustamante MF Agustín-Perez M Cedola F Coras R Narasimhan R Golshan S. Design of an anti-inflammatory diet (ITIS diet) for patients with rheumatoid arthritis. Contemp Clin Trials Commun. (2020) 17:100524. doi: 10.1016/j.conctc.2020.100524 32025586 PMC6997513

[8] Dragan S Șerban MC Damian G Buleu F Valcovici M Christodorescu R. Dietary patterns and interventions to alleviate chronic pain. Nutrients. (2020) 12:2510. doi: 10.3390/nu12092510, PMID: 32825189 PMC7551034

[9] Walsh TP Arnold JB Evans AM Yaxley A Damarell RA Shanahan EM. The association between body fat and musculoskeletal pain: a systematic review and meta-analysis. BMC Musculoskelet Disord. (2018) 19:233. doi: 10.1186/s12891-018-2137-0, PMID: 30021590 PMC6052598

[10] Heneka MT Carson MJ El Khoury J Landreth GE Brosseron F Feinstein DL. Neuroinflammation in Alzheimer's disease. Lancet Neurol. (2015) 14:388–405. doi: 10.1016/S1474-4422(15)70016-5, PMID: 25792098 PMC5909703

[11] Silva AR Bernardo A de Mesquita MF Vaz Patto J Moreira P Silva ML. A study protocol for a randomized controlled trial of an anti-inflammatory nutritional intervention in patients with fibromyalgia. Trials. (2021) 22:198. doi: 10.1186/s13063-021-05146-3, PMID: 33743794 PMC7944600

[12] García-Montero C Fraile-Martínez O Gómez-Lahoz AM Pekarek L Castellanos AJ Noguerales-Fraguas F. Nutritional components in Western diet versus Mediterranean diet at the gut microbiota-immune system interplay. Implic Health Dis Nutr. (2021) 13:699. doi: 10.3390/nu13020699, PMID: 33671569 PMC7927055

[13] Statovci D Aguilera M MacSharry J Melgar S. The impact of Western diet and nutrients on the microbiota and immune response at mucosal interfaces. Front Immunol. (2017) 8:838. doi: 10.3389/fimmu.2017.00838, PMID: 28804483 PMC5532387

[14] Aryaeian N Sedehi SK Khorshidi M Zarezadeh M Hosseini A Shahram F. Effects of hydroalcoholic extract of Berberis Integerrima on the anthropometric indices and metabolic profile in active rheumatoid arthritis patients. Complement Ther Med. (2020) 50:102331. doi: 10.1016/j.ctim.2020.102331, PMID: 32444035

[15] El-Banna HS Gado SE. Vitamin D: does it help Tregs in active rheumatoid arthritis patients. Expert Rev Clin Immunol. (2020) 16:847–53. doi: 10.1080/1744666X.2020.1805317, PMID: 32783547

[16] Gioia C Lucchino B Tarsitano MG Iannuccelli C Di Franco M. Dietary habits and nutrition in rheumatoid arthritis: can diet influence disease development and clinical manifestations? Nutrients. (2020) 12:1456. doi: 10.3390/nu12051456, PMID: 32443535 PMC7284442

[17] Turesson Wadell A Bärebring L Hulander E Gjertsson I Hagberg L Lindqvist HM. Effects on health-related quality of life in the randomized, controlled crossover trial ADIRA (Anti-inflammatory diet in rheumatoid arthritis). PLoS One. (2021) 16:e0258716. doi: 10.1371/journal.pone.0258716, PMID: 34648598 PMC8516209

[18] Vicente BM Lucio Dos Santos Quaresma MV Maria de Melo C Lima Ribeiro SM. The dietary inflammatory index (DII®) and its association with cognition, frailty, and risk of disabilities in older adults: a systematic review. Clin Nutr ESPEN. (2020) 40:7–16. doi: 10.1016/j.clnesp.2020.10.003 33183575

[19] Fox M Knorr DA Haptonstall KM. Alzheimer's disease and symbiotic microbiota: an evolutionary medicine perspective. Ann N Y Acad Sci. (2019) 1449:3–24. doi: 10.1111/nyas.14129 31180143 PMC9495288

[20] Cristofori F Dargenio VN Dargenio C Miniello VL Barone M Francavilla R. Anti-inflammatory and immunomodulatory effects of probiotics in gut inflammation: a door to the body. Front Immunol. (2021) 12:578386. doi: 10.3389/fimmu.2021.578386, PMID: 33717063 PMC7953067

[21] Godos J Currenti W Angelino D Mena P Castellano S Caraci F. Diet and mental health: review of the recent updates on molecular mechanisms. Antioxidants. (2020) 9:346. doi: 10.3390/antiox9040346, PMID: 32340112 PMC7222344

[22] Berk M Williams LJ Jacka FN O'Neil A Pasco JA Moylan S. So depression is an inflammatory disease, but where does the inflammation come from? BMC Med. (2013) 11:200. doi: 10.1186/1741-7015-11-200, PMID: 24228900 PMC3846682

[23] Hewlings SJ Kalman DS. Curcumin: a review of its effects on human health. Foods. (2017) 6:92. doi: 10.3390/foods6100092, PMID: 29065496 PMC5664031

[24] Vellas B Villars H Abellan G Soto ME Rolland Y Guigoz Y. Overview of the MNA--its history and challenges. J Nutr Health Aging. (2006) 10:456–65. PMID: 17183418

[25] Paz García-Portilla M Sáiz PA Díaz-Mesa EM Fonseca E Arrojo M Sierra P. Psychometric performance of the Oviedo sleep questionnaire in patients with severe mental disorder. Rev Psiquiatr Salud Ment. (2009) 2:169–77. doi: 10.1016/S1888-9891(09)73235-5, PMID: 23034346

[26] van Marwijk HW Wallace P de Bock GH Hermans J Kaptein AA Mulder JD. Evaluation of the feasibility, reliability and diagnostic value of shortened versions of the geriatric depression scale. Br J Gen Pract. (1995) 45:195–9. 7612321 PMC1239201

[27] Esteve-Vives J Batlle-Gualda E Reig A. Spanish version of the Health Assessment Questionnaire: reliability, validity and transcultural equivalency. Grupo para la Adaptación del HAQ a la Población Española. J Rheumatol. (1993) 20:2116–22. PMID: 8014941

[28] Sokka T Kautiainen H Hannonen P Pincus T. Changes in health assessment questionnaire disability scores over five years in patients with rheumatoid arthritis compared with the general population. Arthritis Rheum. (2006) 54:3113–8. doi: 10.1002/art.22130, PMID: 17009231

[29] Böhm P Peña-Casanova J Gramunt N Manero RM Terrón C Quiñones-Ubeda S. Spanish version of the memory impairment screen (MIS): normative data and discriminant validity. Neurologia. (2005) 20:402–11. 16217689

[30] Martínez de la Iglesia J Dueñas Herrero R Onís Vilches MC Aguado Taberné C Albert Colomer C Luque Luque R. Spanish language adaptation and validation of the Pfeiffer's questionnaire (SPMSQ) to detect cognitive deterioration in people over 65 years of age. Med Clin. (2001) 117:129–34. doi: 10.1016/s0025-7753(01)72040-4, PMID: 11472684

[31] López Pérez-Díaz AG Calero MD Navarro-González E. Prediction of cognitive impairment in the elderly by analysing their performance in verbal fluency and in sustained attention. Rev Neurol. (2013) 56:1–7. doi: 10.33588/rn.5601.2012281 23250675

[32] Dolsen EA Crosswell AD Prather AA. Links between stress, sleep, and inflammation: are there sex differences? Curr Psychiatry Rep. (2019) 21:8. doi: 10.1007/s11920-019-0993-4, PMID: 30729328 PMC6426453

[33] Karcioglu O Topacoglu H Dikme O Dikme O. A systematic review of the pain scales in adults: which to use? Am J Emerg Med. (2018) 36:707–14. doi: 10.1016/j.ajem.2018.01.008 29321111

[34] Coras R Pedersen B Narasimhan R Brandy A Mateo L Prior-Español A. Imbalance between Omega-6- and Omega-3-derived bioactive lipids in arthritis in older adults. J Gerontol A Biol Sci Med Sci. (2021) 76:415–25. doi: 10.1093/gerona/glaa113, PMID: 32361743 PMC7907486

[35] Christman LM Gu L. Efficacy and mechanisms of dietary polyphenols in mitigating rheumatoid arthritis. J Funct Foods. (2020) 71:104003. doi: 10.1016/j.jff.2020.104003

[36] Trichopoulou A Bamia C Trichopoulos D. Anatomy of health effects of Mediterranean diet: Greek EPIC prospective cohort study. BMJ. (2009) 338:b2337. doi: 10.1136/bmj.b2337 19549997 PMC3272659

[37] González-Domenech PJ Díaz Atienza F García Pablos C Fernández Soto ML Martínez-Ortega JM Gutiérrez-Rojas L. Influence of a combined gluten-free and casein-free diet on behavior disorders in children and adolescents diagnosed with autism spectrum disorder: a 12-month follow-up clinical trial. J Autism Dev Disord. (2020) 50:935–48. doi: 10.1007/s10803-019-04333-1, PMID: 31813108

[38] Farag MA Jomaa SA El-Wahed AA El-Seedi AHR. The many faces of kefir fermented dairy products: quality characteristics, flavour chemistry, nutritional value, health benefits, and safety. Nutrients. (2020) 12:346. doi: 10.3390/nu12020346, PMID: 32013044 PMC7071183

[39] Rondanelli M Faliva MA Barrile GC Cavioni A Mansueto F Mazzola G. Nutrition, physical activity, and dietary supplementation to prevent bone mineral density loss: a food pyramid. Nutrients. (2021) 14:74. doi: 10.3390/nu14010074 35010952 PMC8746518

[40] Fernandes J Fialho M Santos R Peixoto-Plácido C Madeira T Sousa-Santos N. Is olive oil good for you? A systematic review and meta-analysis on anti-inflammatory benefits from regular dietary intake. Nutrition. (2020) 69:110559. doi: 10.1016/j.nut.2019.110559, PMID: 31539817

[41] Gervasi T Barreca D Laganà G Mandalari G. Health benefits related to tree nut consumption and their bioactive compounds. Int J Mol Sci. (2021) 22:5960. doi: 10.3390/ijms22115960, PMID: 34073096 PMC8198490

[42] Davis DW Navalta JW McGinnis GR Serafica R Izuora K Basu A. Effects of acute dietary polyphenols and post-meal physical activity on postprandial metabolism in adults with features of the metabolic syndrome. Nutrients. (2020) 12:1120. doi: 10.3390/nu12041120 32316418 PMC7230938

[43] Nesher G Mates M Zevin S. Effect of caffeine consumption on efficacy of methotrexate in rheumatoid arthritis. Arthritis Rheum. (2003) 48:571–2. doi: 10.1002/art.10766, PMID: 12571869

[44] Rojas-González A Figueroa-Hernández CY González-Rios O Suárez-Quiroz ML González-Amaro RM Hernández-Estrada ZJ. Coffee chlorogenic acids incorporation for bioactivity enhancement of foods: a review. Molecules. (2022) 27:3400. doi: 10.3390/molecules27113400 35684338 PMC9181911

[45] Lemos BS Medina-Vera I Malysheva OV Caudill MA Fernandez ML. Effects of egg consumption and choline supplementation on plasma choline and trimethylamine-N-oxide in a young population. J Am Coll Nutr. (2018) 37:716–23. doi: 10.1080/07315724.2018.1466213 29764315

[46] Philippou E Nikiphorou E. Are we really what we eat? Nutrition and its role in the onset of rheumatoid arthritis. Autoimmun Rev. (2018) 17:1074–7. doi: 10.1016/j.autrev.2018.05.009, PMID: 30213695

[47] Garcia-Gutierrez E Sayavedra L. 3.07—Diet, microbiota and the gut-brain axis In: Glibetic M, editor. Comprehensive gut microbiota. Oxford: Elsevier (2022). 69–83.

[48] Fitzcharles M-A Cohen SP Clauw DJ Littlejohn G Usui C Häuser W. Nociplastic pain: towards an understanding of prevalent pain conditions. Lancet. (2021) 397:2098–110. doi: 10.1016/S0140-6736(21)00392-5 34062144

[49] PuigLRuiz de MoralesJGDaudenEAndreuJLCerveraRAdánA. Prevalence of ten immune-mediated inflammatory diseases (IMID) in Spain. Rev Esp Salud Publica. (2019) 93:e20190301330907380

[50] Guagnano MT D'Angelo C Caniglia D Di Giovanni P Celletti E Sabatini E. Improvement of inflammation and pain after three months' exclusion diet in rheumatoid arthritis patients. Nutrients. (2021) 13:3535. doi: 10.3390/nu13103535, PMID: 34684536 PMC8539601

[51] Hulander E Bärebring L Turesson Wadell A Gjertsson I Calder PC Winkvist A. Proposed Anti-inflammatory diet reduces inflammation in compliant, weight-stable patients with rheumatoid arthritis in a randomized controlled crossover trial. J Nutr. (2021) 151:3856–64. doi: 10.1093/jn/nxab313, PMID: 34587253 PMC8643575

[52] Bruzzese V Scolieri P Pepe J. Efficacy of gluten-free diet in patients with rheumatoid arthritis. Reumatismo. (2021) 72:213–7. doi: 10.4081/reumatismo.2020.1296 33677948

[53] Wu Z Wu B Lv X Xie Y Xu S Ma C. Serumal Lipidomics reveals the Anti-inflammatory effect of flax Lignans and Sinapic acid in high-fat-diet-fed mice. J Agric Food Chem. (2021) 69:9111–23. doi: 10.1021/acs.jafc.0c07291, PMID: 33427466

[54] Rodrigo L Blanco I Bobes J de Serres FJ. Clinical impact of a gluten-free diet on health-related quality of life in seven fibromyalgia syndrome patients with associated celiac disease. BMC Gastroenterol. (2013) 13:157. doi: 10.1186/1471-230X-13-157, PMID: 24209578 PMC3835396

[55] Marum AP Moreira C Tomas-Carus P Saraiva F Guerreiro CS. A low fermentable oligo-di-mono-saccharides and polyols (FODMAP) diet is a balanced therapy for fibromyalgia with nutritional and symptomatic benefits. Nutr Hosp. (2017) 34:667–74. doi: 10.20960/nh.703 28627205

[56] Smedslund G Kjeken I Musial F Sexton J Østerås N. Interventions for osteoarthritis pain: a systematic review with network meta-analysis of existing Cochrane reviews. Osteoarthr Cartil Open. (2022) 4:100242. doi: 10.1016/j.ocarto.2022.100242, PMID: 36475286 PMC9718209

[57] Johansson K Askling J Alfredsson L Di Giuseppe D EIRA study group. Mediterranean diet and risk of rheumatoid arthritis: a population-based case-control study. Arthritis Res Ther. (2018) 20:175. doi: 10.1186/s13075-018-1680-2, PMID: 30092814 PMC6085628

[58] Di Giuseppe D Wallin A Bottai M Askling J Wolk A. Long-term intake of dietary long-chain n-3 polyunsaturated fatty acids and risk of rheumatoid arthritis: a prospective cohort study of women. Ann Rheum Dis. (2014) 73:1949–53. doi: 10.1136/annrheumdis-2013-203338, PMID: 23940215

[59] Navarini L Afeltra A Gallo Afflitto G Margiotta DPE. Polyunsaturated fatty acids: any role in rheumatoid arthritis? Lipids Health Dis. (2017) 16:197. doi: 10.1186/s12944-017-0586-3, PMID: 29017507 PMC5634864

[60] Westerlind H Palmqvist I Saevarsdottir S Alfredsson L Klareskog L Di Giuseppe D. Is tea consumption associated with reduction of risk of rheumatoid arthritis? A Swedish case-control study. Arthritis Res Ther. (2021) 23:209. doi: 10.1186/s13075-021-02583-y, PMID: 34362418 PMC8349003

[61] Papandreou P Gioxari A Daskalou E Grammatikopoulou MG Skouroliakou M Bogdanos DP. Mediterranean diet and physical activity nudges versus usual Care in Women with rheumatoid arthritis: results from the MADEIRA randomized controlled trial. Nutrients. (2023) 15:676. doi: 10.3390/nu15030676, PMID: 36771382 PMC9919932

[62] Bäcklund R Drake I Bergström U Compagno M Sonestedt E Turesson C. Diet and the risk of rheumatoid arthritis - a systematic literature review. Semin Arthritis Rheum. (2023) 58:152118. doi: 10.1016/j.semarthrit.2022.152118 36379128

[63] Shivappa N Godos J Hébert JR Wirth MD Piuri G Speciani AF. Dietary inflammatory index and cardiovascular risk and mortality-a meta-analysis. Nutrients. (2018) 10:200. doi: 10.3390/nu10020200, PMID: 29439509 PMC5852776

[64] Steptoe A Owen N Kunz-Ebrecht S Mohamed-Ali V. Inflammatory cytokines, socioeconomic status, and acute stress responsivity. Brain Behav Immun. (2002) 16:774–84. doi: 10.1016/s0889-1591(02)00030-2, PMID: 12480506

[65] Mathieu S Soubrier M Peirs C Monfoulet LE Boirie Y Tournadre A. A meta-analysis of the impact of nutritional supplementation on osteoarthritis symptoms. Nutrients. (2022) 14:1607. doi: 10.3390/nu14081607, PMID: 35458170 PMC9025331

[66] Sigaux J Mathieu S Nguyen Y Sanchez P Letarouilly JG Soubrier M. Impact of type and dose of oral polyunsaturated fatty acid supplementation on disease activity in inflammatory rheumatic diseases: a systematic literature review and meta-analysis. Arthritis Res Ther. (2022) 24:100. doi: 10.1186/s13075-022-02781-2, PMID: 35526074 PMC9077862

[67] Letarouilly JG Sanchez P Nguyen Y Sigaux J Czernichow S Flipo RM. Efficacy of spice supplementation in rheumatoid arthritis: a systematic literature review. Nutrients. (2020) 12:3800. doi: 10.3390/nu12123800, PMID: 33322318 PMC7764619

[68] Zeng L Yang T Yang K Yu G Li J Xiang W. Efficacy and safety of curcumin and *Curcuma longa* extract in the treatment of arthritis: a systematic review and meta-analysis of randomized controlled trial. Front Immunol. (2022) 13:891822. doi: 10.3389/fimmu.2022.891822, PMID: 35935936 PMC9353077

