# Supplementary material for: The effect of an anti-inflammatory diet on chronic pain: a pilot study
Source: Front Nutr. 2023 Jul 13;10:1205526. doi: 10.3389/fnut.2023.1205526 (PMC10381948; doi:10.3389/fnut.2023.1205526)
Supplement: Supplementary file 1 [file Table_1.DOCX]

**Supplementary materials**

Supplementary Table. Sensitivity, speciﬁcity, and Youden index of different cutoff points for screening sarcopenia in MHD and CKD-NDD patients.

| Patients | Cutoff points | Sensitivity | Specificity | Youden index |
| --- | --- | --- | --- | --- |
| NDD-CKD patients | | | | |
|  | 0 | 100.00% | 0.00% | 0.000 |
|  | 1 | 100.00% | 94.95% | 0.950 |
|  | 2 | 66.67% | 97.98% | 0.647 |
|  | 3 | 33.33% | 97.98% | 0.313 |
|  | 4 | 16.67% | 97.98% | 0.147 |
|  | 6 | 16.67% | 100.00% | 0.167 |
| MHD patients | | | | |
|  | 0 | 100.00% | 0.00% | 0.000 |
|  | 1 | 76.92% | 59.30% | 0.362 |
|  | 2 | 66.67% | 70.93% | 0.376 |
|  | 3 | 56.41% | 81.40% | 0.378 |
|  | 4 | 48.72% | 89.53% | 0.383 |
|  | 5 | 30.77% | 93.02% | 0.238 |
|  | 6 | 15.38% | 93.02% | 0.084 |
|  | 7 | 2.56% | 94.19% | -0.033 |
|  | 8 | 0.00% | 95.35% | -0.047 |
|  | 9 | 0.00% | 98.84% | -0.012 |
|  | 10 | 76.92% | 59.30% | 0.362 |
